# Implementing performance-based financing in peripheral health centres in Mali: what can we learn from it?

**DOI:** 10.1186/s12961-020-00566-0

**Published:** 2020-06-03

**Authors:** Abdourahmane Coulibaly, Lara Gautier, Tony Zitti, Valéry Ridde

**Affiliations:** 1 Miseli Research NGO, Bamako, Mali; 2 Faculty of Medicine and Odonto-Stomatology, Université des Sciences, des Techniques et des Technologies, Bamako, Mali; 3 UMI 3189 Environnement, Santé, Sociétés (CNRS, UCAD, UGB, USTTB, CNRST), Dakar, Sénégal; 4 Department of Sociology, McGill University, Montreal, Canada; 5 Department of Social and Preventive Medicine, University of Montreal, Montreal, Canada; 6 CEPED, Institute for Research on Sustainable Development, IRD-Université de Paris, ERL INSERM SAGESUD, Paris, France; 7 École doctorale Pierre Louis de santé publique: épidémiologie et sciences de l’information biomédicale, Université de Paris, Paris, France

**Keywords:** Implementation, PBF, Mali, CFIR, complex intervention

## Abstract

**Introduction:**

Numerous sub-Saharan African countries have experimented with performance-based financing (PBF) with the goal of improving health system performance. To date, few articles have examined the implementation of this type of complex intervention in Francophone West Africa. This qualitative research aims to understand the process of implementing a PBF pilot project in Mali's Koulikoro region.

**Method:**

We conducted a contrasted multiple case study of performance in 12 community health centres in three districts. We collected 161 semi-structured interviews, 69 informal interviews and 96 non-participant observation sessions. Data collection and analysis were guided by the Consolidated Framework for Implementation Research adapted to the research topic and local context.

**Results:**

Our analysis revealed that the internal context of the PBF implementation played a key role in the process. High-performing centres exercised leadership and commitment more strongly than low-performing ones. These two characteristics were associated with taking initiatives to promote PBF implementation and strengthening team spirit. Information regarding the intervention was best appropriated by qualified health professionals. However, the limited duration of the implementation did not allow for the emergence of networks or champions. The enthusiasm initially generated by PBF quickly dissipated, mainly due to delays in the implementation schedule and the payment modalities.

**Conclusion:**

PBF is a complex intervention in which many actors intervene in diverse contexts. The initial level of performance and the internal and external contexts of primary healthcare facilities influence the implementation of PBF. Future work in this area would benefit from an interdisciplinary approach combining public health and anthropology to better understand such an intervention. The deductive–inductive approach must be the stepping-stone of such a methodological approach.

## Background

Over the past 15 years, performance-based financing (PBF) has attracted attention as a means of achieving specific health objectives more effectively in low-income countries and fragile states [1]. In summarising PBF in both its broadest sense and in the narrow view focused solely on financial incentives, Renmans et al. [2] offer the following definition of PBF-type interventions: “*performance-based financing is a supply-side reform package that is guided towards improved performance (defined a increased predefined services and improved quality measures) by using performance-based financial incentives for health providers (facilities and/or workers)*”. According to this definition, positive performance can be encouraged by linking payments to desired outcomes and by fostering decision-making autonomy and entrepreneurial behaviour at the health facility level [3].

Thirty-two (out of a total of 46) sub-Saharan African countries have tried PBF with the objective of reforming their health system [4]. This intervention is often promoted with the support of certain international actors, in particular the World Bank. Some studies suggest that PBF has had a positive influence on the overall performance of health facilities, particularly on services use [5–7]. Yet, after many experiments, PBF remains controversial in the research community, notably because of discrepancies between the funding allocated and the results obtained [8–10]. Scientific evidence of its effectiveness and efficiency remains limited [11, 12]. In particular, it is criticised for its potential perverse effects, including the fact that it may, in fact, weaken the health system [8] and have unintended effects [13].

The reasons for PBF implementation in Mali were diverse, but the initiative mainly sought to improve health indicators, the management and motivation of healthcare personnel, and access to care [14]. The expansion of PBF is one of the strategic priorities of the Health and Social Development Program 2014–2018 [15]. The PBF project in Mali discussed in this article was funded by the World Bank as part of a larger initiative to strengthen reproductive health. It was implemented in the 10 health districts (HDs) of the Koulikoro region between July 2016 and February 2017. We refer to it here as the ‘second pilot project’; as it followed the implementation of an earlier project in three HDs in this region between 2012 and 2013. This first project was supported by the *Koninklijk Instituut voor de Tropen* (Royal Tropical Institute) and the Dutch development agency *Stichting Nederlandse Vrijwilligers* (we refer to this as the ‘first pilot project’). The designers of the first pilot project called it the ‘pre-pilot’. This project was intended to give concrete expression to the notion of a “PBF *à la malienne*” based on local specificities [16]. A study reported elsewhere highlighted the low level of sustainability of this first project due to, among other things, a lack of sustainability planning [17].

While some research has been conducted on PBF implementation on the African continent [9, 18, 19], studies on the subject are still limited in francophone Africa [20]. The PBF literature remains largely dominated by impact assessments. While these assessments are useful to better understand the effects of PBF on health worker motivation and on health, they do not clearly explain how these effects are produced [3] nor the contexts in which they occur. Some research has shown that analysing the PBF implementation process provides a better understanding of the outcomes achieved [21]. Recent studies have paid more attention to the impacts of PBF on the relationships among actors in the health system and to the contexts and processes that explain whether or not these outcomes are achieved [18, 22–26]. To understand the processes, the contextual characteristics of implementation are essential elements and warrant in-depth analysis [27]. Through this article we aim to help understand the implementation process in community health centres through the use of a qualitative multiple case study approach and of an innovative conceptual framework – the Consolidated Framework for Implementation Research (CFIR).

The research question is the following: How is PBF is implemented and adapted to the socio-political, health and institutional contexts? Our study setting is characterised by multiple features, i.e. health decentralisation (whereby local actors play a leading role), insufficient qualified personnel, low utilisation of services, insufficient access to quality services, poor performance evaluation, and inadequate infrastructure and equipment [28]. In rural areas, the use of family planning services is influenced by male objection according to widespread social norms [29]. One of the three districts selected for data collection participated in a previous PBF project while the other two were utilising it for the first time. Several years before PBF, a previous intervention in the Koulikoro region had implemented an accreditation system with bonuses for the most efficient health centres. Using an innovative, all-encompassing conceptual framework (i.e. the CFIR, see details below), our qualitative research explains how the contextual specificities of the health centres influence the quality of the implementation of PBF by emphasising what makes PBF work in some health centres and not in others. It also highlights the legacy of local history and how the latter might influence implementation processes, thereby reflecting the idea that the past represents a stepping-stone for implementing policies and techniques and securing implementation success [30]. In addition, very few scholars have applied the CFIR to understand the implementation of PBF pilot projects in African countries, particularly in community health centres; our study thus offers to fill this gap. Our findings will be useful for researchers and decision-makers in the current context of the redeployment of PBF in Mali.

## Research methodology

### Conceptual framework

The term ‘implementation’ refers to one or more processes organised in a particular context to help achieve the changes intended by an intervention through the means being deployed [31]. Many theories and conceptual frameworks exist to understand the implementation of interventions. For this study, we chose to use the CFIR, which consolidates key constructs of implementation theories. It was proposed by Damschroder et al. [32] to help assess how effective the implementation of an intervention is in a specific context. The CFIR has proven useful in a wide range of scenarios, including low-income contexts [33]. We chose the CFIR for two reasons. First, it is easy to apply because of its adaptability to the context and research question. Second, it is one of the few tools that can provide a comprehensive view of the intervention within a logically coherent framework.

According to the CFIR, to understand an intervention’s implementation, five ‘domains’ must be studied, namely (1) the characteristics of the intervention; (2) the external context of health facilities; (3) the internal context of health facilities; (4) the characteristics of individuals; and (5) the implementation process. The CFIR consists of 39 constructs and sub-constructs divided among these five domains. The research design we adopted was that of a contrasted multiple case study with several embedded levels of analysis [34]. The cases were community health centres (*Centre de santé communautaire*; CSCOMs), i.e. primary care centres. Data were collected between December 2016 and January 2017 in three of the 10 HDs in the Koulikoro region – HD1, HD2 and HD3. The three HDs were selected on the basis of specific criteria, namely an agricultural site that had experienced being involved in a cash transfer programme for the poorest; a site where it was planned to test a communal mutual insurance program; and a site with an urban character. Of the three HDs selected, only one (HD1) had taken part in the first PBF pilot project in 2012–2013.

Since we were not able to conduct our study in all CSCOMs due to budget and time constraints, we selected four CSCOMs per HD, two from among the highest-performing (CSCOMs '++' and CSCOMs '+' according to performance level) and two from the lowest-performing (CSCOMs '- -' and CSCOMs '-' according to performance level), for a total of 12 CSCOMs within the three HDs [35] (Table 1). The CSCOMs’ performance level was defined on the basis of qualitative and quantitative criteria that emerged from a participatory and consensual process involving the reference health centre (CSREF) teams and a research team composed of the principal investigator (AC) and a doctoral student to support study preparation and to participate in the selection of study sites in view of collecting data for her thesis [35]. For researchers, this offered a timely opportunity to test a model of participatory case selection [36]. This is an innovative approach that makes it possible to legitimise the criteria for site selection beforehand, in particular, the notion of performance by taking into account the health workers’ perspective. The highest-performing CSCOMs were often associated with better involvement of community leaders in activities, greater community mobilisation, greater demographic density, better involvement of the Community Health Association (*Association de santé Communautaire*; ASACO) in activities, dynamic health care personnel, etc. Compared to other CSCOMs, the HD1 CSCOMs had the advantage of benefiting from some of the infrastructure put in place during the first PBF project.

**Table 1:** Number and level of performance of Centres de santé communautaire (community health centres; CSCOMs) by health district (HD)

HD	CSCOM++	CSCOM+	CSCOM- -	CSCOM -	Total
HD1	1	1	1	1	**4**
HD2	1	1	1	1	**4**
HD3	1	1	1	1	**4**
**Total**	**3**	**3**	**3**	**3**	**12**

The four CSCOMs selected in each health district were composed of one urban CSCOM (CSCOM of the district capital) and three rural CSCOMs, with the exception of the Koulikoro HD where there are two urban CSCOMs. Some common characteristics were noted. They relate to the type of the infrastructure (generally including a consultation room, a delivery room, a nursing and a hospitalisation room), the profile of the personnel (most often composed of the technical director of the centre (TDC), nurses, midwives, birth attendants, nurses’ aides, vaccinators, drug depot manager and a hygienist).

The criteria for the inclusion of CSCOMs in the study were defined as follows: have a community health centre status, located in one of the three HDs selected for this study, and be among the CSCOMs classified either as most efficient or less efficient by the end of the selection process. The exclusion criteria were as follows: any CSCOM not located in one of the three HDs selected for this study and any CSCOM not selected by the end of the selection process.

### Description of the intervention

The second PBF pilot project involved a certain number of institutional actors and functions (Table 2).

**Table 2:** Functions and tasks of institutional actors involved in performance-based financing implementation

Institutions	Functions	Tasks
✓ CSCOM	Service provision	- Propose and execute the results plan
✓ CSREF		- Negotiate and sign the contract
		- Produce the services and care
✓ For CSCOMs: Commune and ASACO (pays for the technical services)	Purchasing (contracting)	- Define priorities
		- Negotiate the results plan
✓ For CSREFs: Circle Council		- Negotiate and sign the contract
		- Launch the audit process
		- Purchase the outputs
✓ Project coordination unit/Strengthening Reproductive Health Project	Payment (for outputs produced)	- Pay, after purchaser has signed
		- Ensure availability of funds
✓ HDMT/RHD	Regulation	- Ensure norms and standards are respected – national policy
		- Coach health providers
✓ For CSCOMs: HDMT	Performance auditing of providers (quantity and quality)	- Audit the veracity and reliability of the numbers reported in health centre registers
✓ For CSREFs: RHD		
		- Monitor technical quality
		- Submit a timely audit report to the purchaser
✓ Grassroots community organisation and independent external agency	Cross-auditing of performance at the user level	- Sign a contract with the purchaser
		- Verify whether each person actually received the services
		- Submit a timely audit report to the purchaser
✓ External auditor	Annual external auditing	- Verify the accuracy of the data and expenditures
✓ District management council	Steering committee	- Define programme policies and strategies
✓ National steering committee		- Ex-post monitoring
		- Arbitration in cases of differences of opinions between providers and payers or auditors
✓ Consultancy firm	Technical support	

*ASACO* Association de santé communautaire (community health association), *CSCOM* Community health centre, *CSREF* reference health centre, *HDMT* Health district management team, *RHD* Regional Health Department

At the local level, PBF was implemented in CSCOMs and CSREFs. The quantitative and qualitative results of these providers are evaluated by the HD management team for the CSCOM level and the Regional Health Department for the CSREF level. Once evaluated, results are purchased by the local authorities, which are involved in signing performance contracts with the providers (town hall for the CSCOM and circle council for CSREF). The regulatory function (i.e. checking whether norms and standards are respected) is carried out by the Regional Health Department for the CSREFs and the HD management team for the CSCOM. The funds used to purchase the results are mobilised by the Project Coordination Unit/Strengthening Reproductive Health Project (SRHP). A counter-check is carried out by an independent agency to find out whether patients actually received the health services and their level of satisfaction.

As in any PBF intervention, health facilities are funded based on the purchase of quantity indicators (Table 3) and quality indicators (Table 4). Ten quantitative indicators reflecting major maternal and child health issues were selected for the pilot project in accordance with the priority topics of the World Bank’s SRHP. In addition, three quality domains corresponding to specific scores were covered, namely resources and processes (30%), clinical quality (50%) and user satisfaction (20%).

**Table 3:** Quantity indicators selected for the performance-based financing pilot scheme in Koulikoro

Indicators	Purchase price (Francs CFA)
Prenatal consultation (PNC 4)	3968
Delivery assisted by a qualified professional	1984
Postnatal consultation	661
Use of modern contraception by a woman	2645
Appropriate management of a malaria case in a pregnant woman	1323
Antiretroviral treatment for a pregnant woman (tested HIV positive)	2976
Complete vaccination of a child under 12 months	397
Consultation for a child under 5 years in compliance with integrated management of childhood illness	397
Appropriate management of a malaria case in a child under 5 years	198
Directly observed treatment management of a case of uncomplicated tuberculosis	2645

**Table 4:** Quality indicators by category selected for the PBF pilot scheme in Koulikoro

Category	Content	Weight in calculation of subsidies (value attributed to each category of qualitative indicators)
Resources and processes	- Human resources	30%
	- Infrastructures	
	- Interactions with patients	
	- Hygiene	
	- Governance	
	- Role of the ASACO	
Indicators of clinical quality	- Availability of essential drugs	50%
	- Maternal and neonatal services	
	- Cold chain	
Users’ satisfaction		20%

*ASACO* Association de santé communautaire (community health association)

Typically, quantity indicators are purchased at a fixed price, whereas the payment for quality indicators depends on achieving a minimum target. In the case at hand, after an audit identified any discrepancy between the figures reported by the CSCOMs and the actual provision of services as well as by assessing users’ satisfaction level and verifying the health workers’ quantitative results, an invoice was drawn up and sent via a web portal to a payment agency, which then made the transfer to each CSCOM’s account.

### Data collection tools

Semi-structured interview guides were developed for each category of individual actors interviewed. These various actors included CSCOM personnel, ASACO members (i.e. members of community health associations responsible for managing the CSCOM on behalf of the community), community health workers (i.e. community members who assist qualified health workers mainly through disease prevention and treatment activities within the community), and community leaders who have the power to influence public opinion and members from the commune (local elected officials).

The guides were translated from French into the Bambara language and were then pre-tested. An observation grid was also used. These various guides were developed considering the five domains of the CFIR. Discussions were held beforehand among the co-authors of the article (VR, LG, TZ and VR) to reach a common understanding of the different constructs and sub-constructs.

### Techniques for data collection and sampling

#### Formal and informal interviews

Applying a purposive selection sampling strategy, data were collected from different individual actors involved in PBF implementation in the CSCOMs.

We hired research assistants (*n* = 3) to collect the data. The first author (AC) trained them to use the interview guides. At the start of data collection, AC also provided ‘formative supervision’ by monitoring some interviews conducted by the assistants or by conducting some interviews in their presence. In total, we conducted 161 formal interviews (Table 5) and 69 informal interviews (Table 6) in the three HDs based on the respondent profiles. This informal approach was adopted so the interviewers could collect “*confidences and gossip*” that would have been difficult to access otherwise.

**Table 5:** Distribution of respondents by category and health district, semi-structured interviews

Health districts	Technical directors of the centres	CSCOM personnel	ASACO members	Community leaders	Community workers	Members from the commune	Total
HD1	4	22	8	8	10	4	**56**
HD2	4	18	8	8	8	4	**50**
HD3	4	22	7	10	9	3	**55**
**Total**	**12**	**62**	**23**	**26**	**27**	**11**	**161**

*ASACO* Association de santé communautaire (community health association), *CSCOM* Community health centre, *HD* health district

**Table 6:** Distribution of respondents by category and health district, informal interviews

Health districts	CSCOM personnel	Community workers	Members from the commune	ASACO members	Total
HD1	8	8	2	5	**23**
HD2	8	8	2	5	**23**
HD3	8	8	2	5	**23**
**Total**	**24**	**24**	**6**	**15**	**69**

*ASACO* Association de santé communautaire (community health association), *CSCOM* Community health centre, *HD* health district

Data was collected from these respondents based on their availability [37] and presumed ability to shed light on the situation under study.

CSCOM personnel are often limited in number (five to six staff members). In most cases (10/12), all CSCOM personnel were interviewed. In large CSCOMs and other organisations involved in PBF implementation, we chose to interview the people in charge of or overseeing health activities. By including all these actors, we made sure to consider the diversity of the different actors involved in the implementation of PBF. As the study population was approximately the same size in all districts, we chose a similar sample in each of the three HDs included in the study (69 respondents for the three HDs).

We reached saturation with the total number of interviews conducted in the three HDs. Our deductive approach is based on the application of predetermined codes to the data and, in such an approach, saturation refers to the extent to which predetermined codes or themes are adequately represented in the data [38].

Among CSCOM personnel, the illiterate or least educated are often healthcare assistants or hygienists. We have taken into account the influence of this reality on certain facts, particularly the level of information on PBF, i.e. by comparing this category of staff’s perceptions to those of respondents with higher levels of education, such as nurses, TDCs, depot managers.

At the level of each CSCOM, the first interview was typically conducted with the TDC, who then introduced us to the other actors (health workers, ASACO members, etc.). Research assistants used a recorder to record the interviews as well as personal notebooks to record their own reflections along with data collection. Informal interviews were most often conducted outside the CSCOM, either at the respondent’s house or in another place chosen by the respondent.

#### Observations

Non-participant observations were conducted in the CSCOMs to study interactions between service providers and patients during medical consultations as well as changes made at the managerial level since the launch of the PBF activities (hygiene of premises, data recording procedures). The observation results were recorded in a logbook. The investigators conducted 96 observation sessions for the 12 CSCOMs (HD1 = 32, HD2 = 32, HD 3 = 32), i.e. an average of 8 observations per CSCOM. We considered that this amount of observation sessions were sufficient to reach saturation and address key topics of interest, namely hygiene, the way in which the tools used by the personnel were filled, etc.

### Data coding and analysis process

The researchers classified all interviews conducted and observation notes according to HDs and CSCOMs. All formal interviews, conducted in Bambara, were audio-recorded and then fully transcribed directly into French. Translations from Bambara to French and from French to English were performed by the first and second authors and were further polished by professional translators. The entire dataset (transcripts and notes) was subsequently coded using QDA Miner Lite software. The researchers familiarised themselves with this software beforehand in a 3-day training workshop led by LG. For coding and data analysis, we adopted a deductive–inductive thematic analysis using the CFIR domains, constructs and sub-constructs. These CFIR dimensions guided the definition of the initial codes, subcategories and themes. Coding was performed by two research assistants trained and guided by the first author (AC). Of these two coders, only one took part in the data collection. A coding tree was developed by AC and validated by all the co-authors of the article. The code tree presented the five CFIR domains as general themes while allowing for the inclusion of other themes suggested by the data. An additional file shows this in more detail (see Additional file 1). An interpretive thematic analysis was then applied. Reporting of the findings was entirely guided by the CFIR five domains. We also provided a summary of key findings highlighting critical inter-case comparisons.

At the time of data collection, most of the CSCOMs had started the PBF implementation just 3 or 4 months earlier. As such, it was difficult for the workers to perform certain activities that required more time to become apparent. The following constructs and sub-constructs were not informed by our data: ‘reflection and evaluation’, ‘opinion leaders’, ‘internal leaders formally appointed for implementation’, ‘champions’, ‘evolution’. All these constructs and sub-constructs belong to domain 5 (‘process’) of the CFIR. Although these excluded constructs and sub-constructs are certainly important to describe the implementation process, we were unable to use them due to insufficient empirical data. These sub-constructs were especially related to the theme of stakeholders’ involvement. In making sure that we would still report findings related to this theme, we focused our attention on other forms of actors’ involvement that could be informed by the data, in particular the strategies used to encourage the involvement of various actors or to promote their commitment. Further rationale for excluding these constructs and subconstructs is provided in Appendix in Table 9.

### Ethical considerations

Measures were taken to protect respondents from the potential risks associated with their participation in the study. Every precaution shall be taken to ensure that information concerning them is not divulged, including anonymising of audio recordings and transcripts. Respondents were informed that they could decline to answer certain questions and that they had the right to withdraw from the study when they wished.

## Results

This section is structured as follows: we start with reporting our results following the five CFIR domains, then we offer a summary of inter-case comparisons.

### Characteristics of the intervention

Most respondents (*n* = 117/161) stressed the exogenous origin of the intervention, most often referring to its source of funding. Only the HD1 workers, who had prior experience with PBF, had a more positive perception of it. The results were considered to be proof in themselves:

“*When we look at the results, we can see there really have been changes, especially in the indicators. It’s a CSCOM that’s under-used.*” (TDC, HD1)

The reasons for the positive view of the first pilot project, which at the same time entailed a negative view of the second, had to do with the creation of infrastructures. For most respondents (*n* = 106/161), they were also related to subsidy amounts and the choice of indicators considered more advantageous:

“[In the] *first* [project]*, delivery was paid at 1000 F for birth attendants and 3000 F for nurse-obstetricians, but this time there was nothing for delivery by birth attendants.*” (Birth attendant, HD1)

Other respondents, particularly those from HD3, cited past experiences of CSCOMs being awarded non-PBF-related bonuses, such as the “Ciwara d’Or” (Golden Ciwara). This prize was awarded by the USAID-funded Kènèya Ciwara programme between 2003 and 2008 to reward the best-performing CSCOMs. The quality indicators from this Ciwara programme were used to develop the list of quality indicators for the PBF project.

PBF was seen by most respondents (*n* = 123/161) as an intervention that addressed what local actors considered to be ‘health priorities’. In particular, these included increasing immunisation rates, prenatal consultations (PNC), facility-based deliveries and family planning. However, local personnel were not involved in designing the project. This lack of involvement explained its poor adaptation to the local context, since many respondents (*n* = 86/161) perceived it as having been decided by the World Bank, with no possibility for the actors involved in its implementation to modify the choice of indicators.

“*Often this demotivates us. Because if they say it’s black, then it’s black. It can’t be changed. So, it means we have no impact on that. So we have to live with this*.” (TDC, HD2)

Among the 10 indicators selected, ‘management of mother-to-child transmission of HIV (PMTCT)’ and ‘tuberculosis management’ were considered to be of limited relevance. The workers reported that they had not received sufficient training in these areas and mentioned the limited use of health facilities for these types of consultation.

Malnutrition, curative consultations for adults, geriatric illnesses and childbirth attended by birth attendants were perceived by many respondents (*n* = 123/161) as relevant indicators. Yet, these were not included in the list of selected indicators.

In some cases, health workers were no longer motivated to strive for the centre’s good performance (as intended by the purchase of these indicators) but rather for personal financial gain. According to many respondents (*n* = 83/161), the higher the associated payment, the more relevant and motivating the indicator became:

“*We’re more motivated by indicators that are well paid than by indicators that are not well paid.*” (Nurse, HD1)

With few exceptions, PBF was perceived as a complicated intervention to implement, partly because of data management procedures. The number of working tools, particularly forms and reports, contributed to this complexity:

“*With PBF, entering it in the register isn’t enough, you have to write on the admission form, as required. It’s the reports and procedures that are a bit complicated.*” (Birth attendant, HD1)

Apart from the tools, the complexity of PBF also refers to the sheer number and diversity of actors involved and the distinctions to make between their different roles (e.g. verification and control procedures).

### External context

#### Socio-normative framework and problems experienced

These are situations that are not directly related to PBF but which are in a position to influence its results. Local perceptions of pregnancy resulted in patients’ low attendance at health centres:

“*You call some women to come for PNC, they refuse. They say pregnancy is shameful.*” (Birth attendant, HD1)

Problems experienced by patients and highlighted by health workers were numerous. These included financial difficulties, transportation problems, inadequate information on the negative consequences of certain practices, deficiencies in patient intake and referral, and not enough hospital rooms. Yet, PBF did not offer any solutions to most of these problems.

The external context is also marked by what the actors do and how they behave. This is the focus of the subsection below.

#### Networks of health workers

In the PBF project, the CSCOMs’ quantitative results were audited by the district health team but, due to the project’s limited duration, only one audit cycle could be completed. This short time frame limited the possibility of establishing a system of networks. On the other hand, collaboration between CSCOMs and ASACOs, and between CSCOMs and their communes, was linked to health decentralisation and has existed for a long time. According to many respondents (*n* = 117/161), PBF implementation fostered closer collaboration among these three entities, which worked together to develop the CSCOM’s quarterly results plan in the initial training.

“*We validated the contract together, the objectives were the percentages… We set all that with the staff* [of the CSCOM] *and we tried to find out what the population requires us to do per month; what we can do. This based on each other's ideas, so as to establish the things that we validated together. And we put that in the contract*.” (TDC, HD1)

Quarterly results plans are documents that describe the main obstacles to improving health indicators, the solutions proposed to address them and the means needed to implement those solutions. The quarterly results plans are drawn from the CSCOM’s annual micro plan, which is a kind of business plan that identifies trends in the quantitative and qualitative indicators to be purchased and includes the CSCOM’s projected cash inflows and outflows to purchase these indicators. They contain the technical elements of health planning required to implement the agreements set out in the tripartite contract between the commune, the ASACO and the CSCOM technical team.

The three entities (CSCOM, ASACO and commune) also joined forces to conduct awareness-raising activities in the villages. Additionally, the commune representative was often invited to attend ASACO meetings, and vice versa.

#### A competitive environment

PBF was perceived by most respondents (*n* = 92/161) as establishing a logic of competition because the CSCOMs and even the villages tended to compare one another in terms of performance:

“*When we talk about PBF, the competition becomes tangible. So we need to really emphasise the competition among villages in the* [health] *area. Not only among villages in the area, but also among the CSCOMs. No one wants to come in last.*” (TDC, HD1)

Another respondent concurred, using a militaristic idiom:

“*It’s competition, that’s what it is. In any case, for us, it’s a weapon of warfare*.” (TDC, HD1)

PBF has been added to an already competitive environment. In the Mali setting, competition is justified by pride in being recognised among the best. Competition may materialise at the family, village, inter-village and communal levels. Any economic activity can also become a space for competition (agricultural production, construction of development infrastructures, etc.).

This competitive logic was largely fuelled by the existence of projects such as the “Golden Ciwara” in the past and the “Blue Star”, still in progress, whose approaches greatly value the idea of competition among CSCOMs. Besides the CSCOMs, communes and health areas, this competition played out among ASACOs and even HDs, engaged to improving access, quality and use of high-impact health services as well as household members’ adoption of good maternal and child health practices.

According to many respondents (*n* = 89/161), this competition impelled them to achieve greater efficiency:

“ *…* [if] *there’s competition, everyone tries to save their own heads… So people will make the effort. You’ll find that there are things we don’t think of doing, but as soon as they say this is how it has to be, we’ll scramble to do it, you see.*” (Community leader, HD3)

Concretely speaking, competition results in the frequent introduction of incentives to motivate staff to stay in their current workplace.

“*The housing problem for our workers, if we could get the money, we could build for them and they wouldn’t go anywhere else. If there is competition, if our work is well done, the CSCOM will be one of the best*.” (Cleaning staff, HD3)

#### Influence of public policies

The 2014–2018 Social and Health Development Program (PRODESS III) referred to PBF, providing details on its definition, benefits and functioning [15]. PBF was also featured among the activities planned by the World Bank’s SRHP as a means to strengthen the supply and quality of reproductive health services by improving indicators in this area. Thus, PBF is expected to contribute to the achievement of a range of objectives of each of these programmes as well as those of the 2014–2018 National Reproductive Health Strategic Plan.

### Internal context

#### Information dissemination about PBF

TDCs and colleagues who had attended the initial training subsequently organised briefing sessions where they shared information on PBF functioning with the other workers who had remained on site. The briefings provided an opportunity to communicate the objectives contained in the results plans. However, some respondents, particularly the cleaning staff, felt that they had simply been neglected and had not benefited from this information sharing.

They felt that the briefings were validation sessions of what the CSCOM representatives had already decided, rather than real discussions: “*It’s the leader doing this and then informing us*” (Birth attendant, HD1). In some cases, the development of the performance contract objectives was largely dominated by the TDC, who involved few other staff. The performance contracts set out, on a quarterly basis, the commitments of the signatory parties and the projected results. At the CSCOM level, the contract was signed by the TDC, the chairman of the ASACO, and the commune mayor.

During the meeting, information was given on PBF tools:

“*The TDC brought us together. He had some files to explain how the work on PBF could be done. The part we should sign, we signed.*” (Drug depot manager, HD1).

In some cases, informal discussions with the TDC improved staff’s sentiment of being better informed about PBF.

#### Convergence between professional standards valued and PBF- standards valued

PBF encourages the development of team spirit within health teams. PBF is intended to ensure equity among workers by paying each according to their performance. The values and professional standards identified by the majority of respondents (*n* = 97/161), are illustrated by the following excerpts from interviews:

“*It produces cohesion because we collaborate with each other*” (Commune member, HD3)

“*When merit is rewarded, I think everyone will apply themselves to quality. That’s it, really.*” (Drug depot manager, HD3)

“*It will help us educate ourselves on the idea of work, that is, being punctual, being present every day, giving ourselves autonomy in our work, and empowering people.*” (Midwife, HD1)

#### Forms of commitment to PBF

Commitment was expressed in the TDC’s efforts to inform other staff members of the importance of PBF:

“*I remind the staff that they need to do their job well, and I remind them of the importance of implementing PBF*.” (TDC, HD1)

Some ASACOs agreed to invest in infrastructure renovations, while others made efforts to reinforce human resources. Commitment was also reflected in regular visits by the ASACO chairman or members to the CSCOM to observe the progress of activities:

“*If the* [ASACO] *chairman is seen here every morning, it’s because we’re respecting our commitments to PBF.*” (Vaccinator, HD2)

One respondent from HD1 noted that other ASACO members were visiting the CSCOM much more regularly since the launch of the intervention. Every staff member had signed a personal commitment form. The TDCs asked that this form be posted in front of the offices so that everyone’s commitment would be visible. In some cases, this commitment depended on the degree of motivation. For example, for birth attendants who did not receive premiums for deliveries, the commitment had to be put into perspective:

“*I myself am a birth attendant. What I’ve noticed, in my view, in this new PBF, is that it’s birth attendants who come last. That’s what I see.*” (Birth attendant, HD1)

One respondent saw PBF as creating a situation of unfairness among workers, with the most qualified being treated better than the others:

“*No, they’re not rewarding everyone’s work, just those who are qualified professionals.*” (Community leader, HD3)

#### The influence of local legacies

Participation in the first PBF project, as is the case in the HD1, was used as a reason that justified the CSCOM’s readiness to implement PBF:

“*As long as there is this old system, the second system cannot fail and can work well*” (Member from the commune, HD1). The majority of HD1 respondents (*n* = 43/56) said that their centre was ready to implement the new PBF project based on the fact that there was a system put in place during the previous PBF that could well contribute to its effectiveness: “*The materials we had, we can work with that*” (Hygienist, HD1). In addition, some CSCOMs had benefited from the interventions and support provided by NGOs such as Marie Stopes International and *Kèneya Ciwara* through a CSCOM accreditation programme as well as the AMCP ALIMA with actions in the field of nutrition. According to several respondents (*n* = 89/161), these legacies from the past are an asset for the CSCOMs engaged in strengthening their equipment training and organisational capacities:

“*They have transferred skills and that is the main thing. When skills are transferred, even if you leave, the staff can work after you.*” (TDC, HD3)

### Characteristics of individuals

Those interviewed in the centres had been quite receptive to the change proposed by PBF (*n* = 70/74). News of the arrival of PBF generated enthusiasm. This positive response often stemmed from their having heard about the successful implementation of the first pilot project. For many respondents (*n* = 89/161), the enthusiasm expressed at the time of introduction dissipated due to the lack of any PBF-specific supervision once the project was under way. In some cases, the good practices generated by PBF, such as the introduction of attendance lists, quickly disappeared:

“*We were expecting supervision and then we're going to give bonuses, but this hasn't been done so far, which is why we have abandoned the attendance book*.” (Nurse, HD3)

Data analysis showed that a number of respondents were unable to provide detailed information on certain aspects of the PBF scheme, in particular relating to the calculation of staff bonuses and the procedures for the payment of bonuses and subsidies. Among the information most mastered by the respondents were the objectives of the intervention, with a focus on motivation and payment according to the effort made. The information on PBF was best mastered by the most qualified agents.

Most of the reasons given by respondents to explain their positive attitudes towards PBF were based on their perception that the initiative’s objectives incorporated values they defended (work well done, merit rewarded, consensus, cohesion, etc.):

“*Because I've always said. It's so very much about rewarding merit.*” (TDC, HD3)

However, the conditions defined for financial incentives were a source of frustration for some workers, especially for the less qualified, as noted above in the case of birth attendants. Some respondents expressed a feeling of relative satisfaction with PBF, while also showing some form of concern:

“ *… We were all happy and worried at the same time, because it’s a new thing for us.*” (TDC, HD3)

### Implementation process

Within the PBF framework, the CSCOMs’ activities were planned on a quarterly basis. The only intervention outcome plans were developed during the initial training. At the time of our data collection, CSCOM personnel were focused more on making changes to maximise their individual premiums. The schedules for the various activities were rarely respected. Certain activities, such as staff evaluations, could not be carried out in some cases for lack of time. In fact, the initial time frame allotted for project implementation (8 months) was reduced to 6 months due to numerous problems (2012 political crisis, recruitment of the implementing agency, management problems, etc.). Initially, it was announced that premiums would be paid on the basis of two working quarters. In the end, workers received premiums for only one quarter, with payment delays of up to 6 months in some cases.

Despite the problems encountered by health teams, significant changes appeared to have been made in several areas. Schedules for outings in outreach strategies (teams going to villages to conduct health activities) were modified; in particular, the duration of outings was increased. Some CSCOMs decided to strengthen the vaccination team by recruiting again. This trend of recruiting additional staff was not observed in CSCOMs with low performance levels. To reduce the cost of prescriptions for children, in accordance with a quality standard imposed by the PBF project, some ASACOs negotiated with health workers to encourage them to talk with patients with a view to assessing their financial capacity and then issuing prescriptions based on their situation. This was done so that premiums would not be negatively affected due to non-compliance with this standard. One of changes mentioned most often by respondents with regard to the on-call system was that, with the introduction PBF, skilled personnel (nurse, nurse-obstetrician, midwife, TDC) became much more present during childbirths than before. In accordance with the new PBF instructions, when childbirth cases arrived in the absence of skilled personnel during on-call periods, the birth attendants called them to attend the childbirths, unlike the pre-PBF period when they could attend these deliveries on their own. For eligible staff, the objective of such a measure was to earn more premiums related to these deliveries.

Intake evolved from being ‘non-welcoming’ to showing greater availability to patients. The measures taken in the area of intake were verbal instructions given to agents to welcome patients and changes in the reception system. In most cases, both measures were introduced. Some CSCOMs increased the number of consultation rooms so as to avoid queues. Under PBF, the intake system included referring patients to the services they needed. In the CSCOMs classified as low-performing, measures to improve intake more often took the form of verbal instructions. Referral of patients to services that meet their needs is a new initiative in many centres. While the measure existed before PBF, it has been strengthened with the implementation of the project.

In the area of hygiene, the PBF project compelled almost all the CSCOMs to adopt new rules on cleaning. These rules refer to the quantity and quality of cleaning carried out. They have made it possible to make changes in the frequency of facility cleaning, in the number of surfaces to clean and in the degree of cleanliness of the spaces cleaned. Cleaning teams were also reinforced. According to most respondents (*n* = 126/161), the rate of cleaning increased considerably:

“*Cleaning is done every morning, first thing. Often when the hygienist has finished the job, we do the rest after the childbirth, and we also do the cleanup.*” (Nurse, HD1)

PBF also resulted in a better drug management system. Drug depot managers regularly checked the stock to avoid stock shortages. Ordering procedures were streamlined and orders were sent fairly quickly as soon as the stock decreased. Monthly drug inventories were carried out by the ASACOs and done regularly, according to the depot managers. All drug depot managers (*n* = 12/12), reported that they became more diligent in filling out drug management tools

“*Since the new PBF started, I fill out these documents every morning.*” (Drug depot manager, HD1)

### Inter-case comparisons summary

In this section, we offer a summary of the findings, highlighting the similitudes and differences across the different cases. The data revealed differences between high-performing and low-performing CSCOMs in the approach adopted for PBF implementation (Table 7). These significant differences were seen in the ‘implementation climate’ and ‘readiness for implementation’ constructs related to the internal context (CFIR domain 3) and in the ‘engaging’ construct related to the implementation process (CFIR domain 5).

**Table 7:** Comparison of high- and low-performing CSCOMs by CFIR domains and constructs

Domain	CFIR construct	High-performing CSCOMs	Low-performing CSCOMs
Internal context	**Implementation climate:**	Perception that the CSCOMs had prepared well for PBF implementation	Staff of some CSCOMs reported that the conditions required to start the PBF were not met due to lack of equipment and infrastructure
	- Tension around change		
	**Implementation climate:**	Objectives set out in the contract were, in many cases, discussed before being ratified	The objectives were hardly discussed with the rest of the staff
			Results plans were seldom shared with the rest of the staff
	- Objectives and feedback	Briefing sessions were used to communicate the objectives in the results plans	
	**Implementation climate:** - A learning environment	Awareness of being a single team in which each member is personally responsible for the outcomes Stronger collective commitment	
	**Readiness for implementation:**	TDCs explained the data on the importance of PBF to the rest of the staff to motivate them	Weak leadership of TDCs; often conflictual interactions with the ASACO
	- Leadership engagement		
Process	**Engaging**	Many awareness-raising activities conducted by a team consisting of the TDC, the commune mayor and the ASACO chairman	TDCs led most of the awareness-raising sessions on their own

*ASACO* Association de santé communautaire (community health association), *CFIR* Consolidated Framework for Implementation Research, *CSCOM* Community health centre, *PBF* performance-based financing, *TDC* Technical Director of the Centre

The best performing CSCOMs are characterised by a better flow of information between the TDCs and the rest of the team, a predominance of team spirit and more initiatives to encourage the population to use services (Table 7).

Analysis of the data also revealed similarities and differences among HDs (Table 8). The specific features refer to three of the five CFIR domains, namely internal context, characteristics of individuals and implementation process.

**Table 8:** Summary of PBF implementation results in the three health districts according to the five CFIR domains

CFIR domains	Similarities among districts	Specific features		
		HD1	HD2	HD3
1. 1. 1. **Characteristics of the intervention**	- PBF perceived as a foreign intervention	NA		
	- Difficulty citing the name of the funder			
	- Perceptions of a complicated intervention			
2. 1. 1. 1. 1. **External context**	- Late recourse to care	NA		
	- Insufficient vaccination coverage of children			
	- PBF network not well developed among the health personnel involved in its implementation			
	- Presence of NGOs that could contribute to the achievement of PBF objectives			
	- Implementation of the Social and Health Development Program			
3. 1. 1. 1. **Internal context**	- Strong correlation between level of information and level of education	Experience of having been involved in implementing a previous PBF project	First experience of involvement in a PBF project	First experience of involvement in a PBF project
	- Personnel focused on financial incentives			
	- Appearance of different forms of engagement towards PBF			
	- Perceptible frustration among staff with less training			
4. 1. **Characteristics of individuals**	- Personnel receptive to change	Arguments in favour of PBF were based on the success of the previous PBF project	Arguments in favour of PBF were based on the values attributed to it and on rumours about the PBF project	Arguments in favour of PBF were based on the values attributed to it and on rumours about the PBF project
	- Perceived link between PBF objectives and professional values held by workers			
5. 1. 1. 1. 1. **Implementation process**	- Schedule of planned activities not respected	Greater proficiency with PBF tools and content	Low proficiency with PBF tools and content	Low proficiency with PBF tools and content
	- Reforms implemented to maximise results			
	- Increased connivance among workers			
	- Increased presence of skilled personnel during on-call shifts			
	- Recruitment of new personnel by some CSCOMs			

*CFIR* Consolidated Framework for Implementation Research, *CSCOM* Community health centre, *HD* health district, *NA* not applicable, *PBF* performance-based financing

## Discussion

With the CFIR, which is increasingly being used in low- and middle-income countries [39], we were able to conduct an in-depth contextual analysis, reflecting in particular on the internal and external contexts of the PBF intervention. There is a recurrent conclusion in the literature that context has been neglected in research aimed at developing and evaluating population health interventions [40]. In sub-Saharan Africa, any analysis of the implementation of public health interventions must consider context [41]. The role of actors is also crucial in the implementation and ownership process of a reform, where the pluralism of norms and their instability often forces social actors to make multiple adjustments [42]. Implementation may also be influenced by the fact that the practical norms and professional cultures that govern the actual behaviour of employees are far removed from official norms. In addition, public goods and services are co-provided by a series of different actors and institutions, with little coordination among them – this feature may also shape implementation processes [43].

In the context we studied, our attention was drawn to certain constructs related to the internal context. The differences between high-performing CSCOMs and low-performing CSCOMs were highlighted when applying some constructs and sub-construct of this domain, which also reflected attitudes and practices that were especially evident in the intervention start-up phase. These were the construct of ‘implementation climate’ and the sub-construct ‘leadership engagement’ (under the construct ‘readiness for implementation’). In addition, our analysis of the issue of leadership showed this to be a factor that promoted good performance. As such, CSCOMs led by a TDC with strong leadership achieved more reforms in this PBF intervention. A study conducted in Burkina Faso in district hospitals highlighted that health centre managers’ leadership and vision were essential ingredients for effective health action, particularly in contextualising national strategies and taking patients’ concerns into account [44].

Our data underscore a strong link between leadership and engagement. In the more successful CSCOMs, managers’ commitment fostered staff commitment. In these CSCOMs, the start-up phase of the intervention was marked by multiple efforts from TDCs to explain to the rest of the staff the importance of PBF in order to motivate them. Recent research has shown the importance of leadership styles (directional or participatory) in determining health workers’ motivation for PBF in Mali [45].

PBF is an intervention that, like any other, requires both individual and collective commitment on the part of the actors involved. Individual commitment was theoretically manifested in each CSCOM employee’s signing of a commitment form. Collective commitment was expressed as a heightened spirit of initiative to improve the chances of gaining more subsidies. This spirit of initiative was seen more often in the high-performing CSCOMs than in the others, which performed poorly in terms of the redesign of data collection tools, service organisation, measures to improve patient intake and better hygiene.

The literature identifies several contextual factors that could potentially explain the difference in performance among facilities involved in PBF implementation [46–48]. In particular, authors point to the uncertainty surrounding the payment of premiums (e.g. late payment), communication among stakeholders, confidence in the performance measurement instrument methodology, the health workers’ understanding of how PBF works and the role of facility managers (management skills).

PBF can affect health workers’ motivation in a variety of ways, and often in ways that extend far beyond the direct effects of financial rewards for individuals [26]. One study in Nigeria showed that health workers’ motivation and performance were reduced by uncertainty about obtaining the incentive and inadequate infrastructure [49]. Conversely, a good understanding of the system among health workers and strong management skills are likely to improve motivation and performance, just as reducing delays in incentive payments, communicating effectively and strengthening health workers’ understanding of the PBF system are likely to produce better results in pay-for-performance programmes. Another study conducted in Sierra Leone found that implementation deficiencies, such as late payment and access difficulties, posed a series of problems that limited the motivational effects of incentives [50].

The analysis of our data showed the PBF project also generated uncertainty and frustration among those involved in its implementation. These had to do, among other things, with the long delay in starting activities and the lack of consideration for deliveries carried out by birth attendants, particularly for those in HD1, who had experience with the first pilot PBF project in which these deliveries had been taken into account. These situations created a certain scepticism initially, even though the majority of health workers expressed buy-in for the intervention. In the interviews, the PBF remained an important source of motivation for the health workers. Their motivation was linked not only to subsidies but also to improved working conditions (acquisition of equipment, infrastructure) and better system governance (rewarding merit). As such, PBF stimulated an interactive relationship between internal and external motivational factors related to responsibility, achievement and recognition, thus increasing perceived motivation [51].

Some studies have strongly underscored the importance of PBF-related motivation associated with work environments, including systematic supervision and availability of essential drugs [23]. In Benin, a contextual analysis of the implementation of two different PBF models showed that, in this field, there is no rigid and standardised model because each context dictates its specific features [52]. Given the specificities of contexts and characteristics, the ‘same’ project will be ‘different’ in each facility, i.e. some mechanisms may work in one context and not in another, to the point where it becomes possible to identify features specific to each PBF system [2].

It is important to note that PBF does not operate in a vacuum within health systems. Some studies have underscored the complexity of these interventions, highlighting the notion that an intervention can be seen as a critical event in a system’s history, resulting in the emergence of new structures of interaction and new shared meanings [53]. The complexity of social interactions is a property of both the intervention and the context [54]. Often, both CSCOM workers and the general population perceived PBF as just another development project like so many others they had seen over time, as noted regarding PBF in Burkina Faso [25]. In this context, projects come and go, but to the actors involved, they all look the same [55]. Indeed, they are often implemented in an environment that has undergone many previous interventions that have left their traces. Such local history necessarily structures present behaviours, at least in part [56]. This observation reflects the notion of ‘path dependency’, in which each new reform is defined in reference (positive or negative) to a set of past policies [57]. The logic that generally prevails among project designers is to consider the story as starting with their own project, and then systematically underestimating all that has been done before and overestimating their project’s impact. PBF is no exception to this rule, as the payment of performance-based premiums that is at the heart of this intervention is a common practice. In the past, some projects also offered premiums linked to health facilities’ overall performance. As a public policy, PBF has been analysed at the national level based on an accumulation of experiences in which local actors have played a particularly important role as Ma-Nitu et al. [58] have suggested in other African contexts.

The CFIR also enabled us to reflect on practices to encourage uptake of the intervention. PBF is anchored in comprehensive standards for specific reforms – renewal of public management structures, separation of functions (buyers–providers), strengthened supervision, greater autonomy of service providers and enhanced effectiveness of information systems [59]. This research highlights how uptake is constructed through the interplay of multiple actors. The qualitative approach allowed us to go beyond the mechanical application of the CFIR constructs and to give the analysis a less schematic character by showing, in a concrete way, how social actors co-construct the reality of the implementation of PBF. Indeed, analysing the results through the lens of the CFIR domains and constructs, in particular domains 1 (characteristics of the intervention), 2 (internal context) and 3 (external context), revealed useful information on the ways in which local actors adapted PBF and shaped it to their own logic. Such uptake occurs in a system of norms and values that can influence the way it manifests. We observed, for example, that the PBF implementation’s external context was marked by attitudes and practices that could impede services use. An example of this was the sense of shame associated with pregnancy that prevented many women from going to the health centre for PNC. Along these lines, some studies have noted that the social context can directly affect the attainment of PBF objectives if behaviours advocated by this intervention are not socially or culturally anchored [60]. Others have shown that how equity is conceived in a given context can influence the acceptability of meritocratic payment systems [61].

Overall, certain ‘visible’ changes marked the launch of the PBF project, notably the various changes made to the on-call system, vaccination outings, patient intake and hygiene. These reforms show that PBF implementation can have an impact on the health system, particularly on service delivery, human resources and governance [3].

Some studies have shown that giving specific information to the community increases the effectiveness of participation [62]. This information-sharing approach corresponds to the theory of change centred on responsibility towards the community, as opposed to the theory of change centred on responsibility towards purchasers of services [2]. The case presented in this study belongs to the latter theory of change, notably because what the health workers primarily retained from PBF was that they should receive premiums and that these were linked to achieving performance indicators. Their statements reflected the fact that they had very little understanding of the complexity of PBF functioning; a similar observation was made in Burkina Faso [25].

In demonstrating the influence of context on the implementation of PBF through the inter-case comparative approach, our research emphasised that, in a high performance context, health facilities’ human resources are better organised to implement PBF than in a low performance context.

Due to subsequent delays in the implementation of the pilot scheme and the very short time for implementation, the present research could only study the start-up phase. This represents a limitation and long-term trends are not known. At the same time, this situation emphasises the need to adapt the CFIR according to the stages of a programme’s implementation (start-up, mid-term, post-implementation) and more broadly to the context of the study [39].

In addition, the results of our research contribute to a better understanding of the prerequisites that must be in place to support the achievement of the objectives identified in the results plans.

## Conclusion

In this qualitative study, using the CFIR enabled to better understand the process of implementing PBF based on the CFIR’s five domains – (1) the characteristics of the intervention, (2) the context external to the health facilities, (3) the context internal to the health facilities, (4) the characteristics of the individuals, and (5) the implementation process. We have particularly emphasised the role of the contextual specificities of the health facilities. The external and internal contexts, in particular, played a decisive role in the implementation process. The leadership and commitment of the TDCs are much more pronounced in the high-performing CSCOMs than in the lower-performing CSCOMs. In addition, the most significant differences between well-performing and less performant CSCOMs are to be found in the internal context (constructs ‘implementation climate’ and ‘readiness for implementation’) and the implementation process (under construct ‘engaging’). The study emphasised that the implementation of the PBF does not result in a mechanical application of the official standards and that these standards are always modulated according to the context of implementation. Contexts indeed play a determining role in implementation effectiveness.

The CFIR is a rich conceptual framework that makes it possible to conduct a sufficiently detailed contextual analysis, but whose dimensions are not always fully in line with our own reflections. As we progressed through our application of CFIR, we deliberately opted for an ‘open approach’, whereby we integrated notions that seemed relevant for the analysis of some constructs, without betraying their original definition. This back and forth between original meaning and ‘extended’ meaning makes the CFIR a suitable tool for analysing the implementation of an intervention.

### Supplementary information

**Additional file 1.**

## Data Availability

Not applicable.

## Appendix

**Table 9:** Reasons for non-inclusion of certain constructs and sub-constructs

Constructs and sub-constructs	Reasons for non-inclusion
Opinion leaders	Opinion leaders, such as imams, politicians and presidents of women’s associations and youth associations, were not involved in the implementation of the project
Leaders formally appointed for the implementation	In the three districts, no community health centre (CSCOM) had formally appointed any agents for performance-based financing implementation
Champions	The implementation period was not long enough to allow for the emergence of champions
Reflection and evaluation	In the start-up phase, no reflection or evaluation had yet been carried out
Evolution	At the start of the intervention, no data was available to monitor whether the activities were progressing according to the implementation plans

## Abbreviations

ASACO: Association de santé communautaire (community health association)
CFIR: Consolidated Framework for Implementation Research
CSCOM: Centre de santé communautaire (community health centre)
CSREF: reference health centre
HD: health district
PNC: prenatal consultation
PBF: performance-based financing
RHD: Regional Health Department
SRHP: Strengthening Reproductive Health Project
TDC: Technical Director of the Centre
