# Supplementary figures and images for: Implementing performance-based financing in peripheral health centres in Mali: what can we learn from it?
Source: Health Res Policy Syst. 2020 Jun 3;18:54. doi: 10.1186/s12961-020-00566-0 (PMC7268714; doi:10.1186/s12961-020-00566-0)

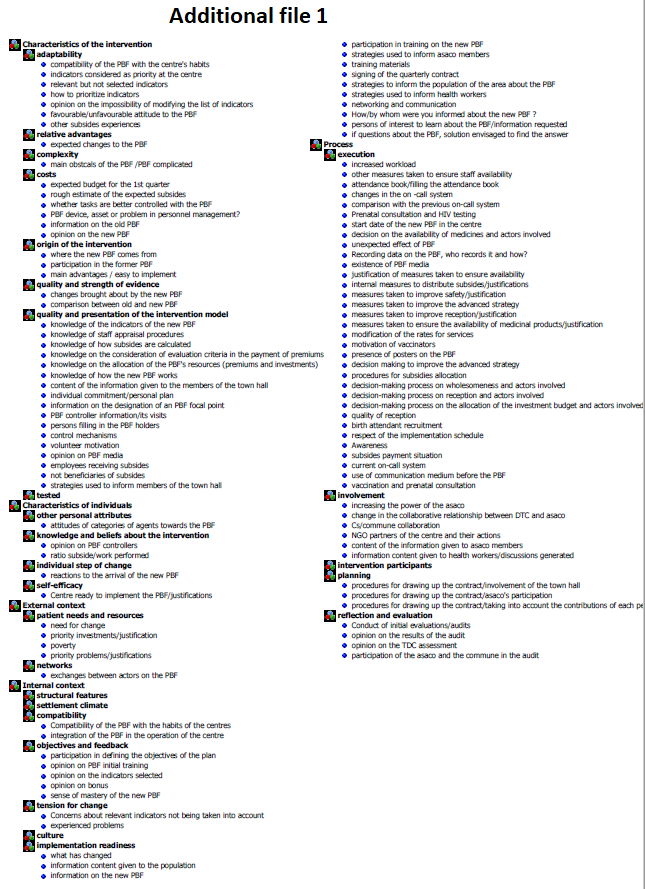

Supplement: Supplementary file 1 — Additional file 1. [file 12961_2020_566_MOESM1_ESM.docx]
